# Epidemiology of Tick-Borne Borreliosis in Morocco

**DOI:** 10.1371/journal.pntd.0001810

**Published:** 2012-09-13

**Authors:** Georges Diatta, Yassine Souidi, Laurent Granjon, Céline Arnathau, Patrick Durand, Gilles Chauvancy, Youssouph Mané, M'hammed Sarih, Driss Belghyti, François Renaud, Jean-François Trape

**Affiliations:** 1 Institut de Recherche pour le Développement, Laboratoire de Paludologie et de Zoologie Médicale, UMR 198, BP 1386, Dakar, Senegal; 2 Faculté des Sciences de Kénitra, Université Ibn Tofail, Kénitra, Morocco; 3 Institut Pasteur du Maroc, Casablanca, Morocco; 4 Institut de Recherche pour le Développement, Centre de Biologie et de Gestion des Populations, UMR 22, BP 1386, Dakar, Senegal; 5 Institut de Recherche pour le Développement, UMR 5290 MIVEGEC IRD/CNRS/UM, BP 64501, Montpellier, France; University of California Davis, United States of America

## Abstract

**Background:**

The presence in Morocco of Argasid ticks of the *Ornithodoros erraticus* complex, the vector of tick-borne relapsing fever (TBRF) in North Africa, has been known since 1919, but the disease is rarely diagnosed and few epidemiological data are available.

**Methodology/Principal Findings:**

Between 2006 and 2011, we investigated the presence of *Ornithodoros* ticks in rodent burrows in 34 sites distributed across Morocco. We also collected small mammals in 10 sites and we investigated TBRF in febrile patients in Kenitra district. The prevalence of *Borrelia* infections was assessed by nested PCR amplification in ticks and the brain tissue of small mammals, and by evaluation of thick blood films in patients. A high proportion of burrows were infested with ticks of the *O. erraticus* complex in all regions of Morocco, with a mean of 39.5% for the whole country. *Borrelia* infections were found in 39/382 (10.2%) of the ticks and 12/140 (8.6%) of the rodents and insectivores studied by PCR amplification, and 102 patients tested positive by thick blood film. Five small mammalian species were found infected: *Dipodillus campestris*, *Meriones shawi*, *Gerbillus hoogstrali*, *Gerbillus occiduus* and *Atelerix algirus*. Three *Borrelia* species were identified in ticks and/or rodents: *B. hispanica*, *B. crocidurae* and *B. merionesi*.

**Conclusions/Significance:**

Tick populations belonging to *O. erraticus* complex are widely distributed in Morocco and a high proportion of ticks and small mammals are infected by *Borrelia* species. Although rarely diagnosed, TBRF may be a common cause of morbidity in all regions of Morocco.

## Author Summary

In North Africa, Argasid ticks of the *Ornithodoros erraticus* complex are the only known vector of *Borrelia* infections causing tick-borne relapsing fever (TBRF) in humans. There is limited data on vector distribution, the animal reservoir of the disease has never been investigated, and there is no published data on TBRF patients. Our aim was to systematically investigate the distribution of *O. erraticus* s.l. in most regions of Morocco, to measure the proportion of infected ticks, to identify small mammalian species that act as potential reservoir, and to analyze data on TBRF patients fortuitously collected during a malaria eradication program. Our study shows that a high proportion of rodent burrows are colonized by vector ticks in all regions of Morocco from the Atlantic Sahara to the Mediterranean coast. We identified three *Borrelia* species in ticks and/or small mammals: *B. hispanica*, *B. crocidurae* and *B. merionesi*. We report five species of small mammals found infected for the first time. Our analysis of 102 TBRF patients shows that the disease is strictly seasonal in northwestern Morocco with a maximum incidence during summer. We believe that TBRF, although rarely diagnosed, is a common cause of morbidity in all regions of Morocco.

## Introduction

Tick-borne relapsing fever (TBRF) due to *Borrelia* infections transmitted by Argasid ticks of the genus *Ornithodoros* is a major cause of disease in several regions of Africa. In Senegal, the incidence of TBRF in rural communities ranges between 4% and 24% each year [1] and in villages of Tanzania up to 38% of infants are infected before reaching one year of age [2]. Three different *Ornithodoros*-*Borrelia* systems have been described in Africa [3], [4]. In Eastern and Southern Africa, TBRF is due to *B. duttoni* with *O. moubata* and *O. porcinus* as vectors; in West Africa and the most arid parts of North Africa, TBRF is due to *B. crocidurae* with *O. sonrai* as the vector; whereas in coastal areas of North Africa, TBRF is due to *B. hispanica* with *O. erraticus* as the vector [3]–[7]. Both in West and North Africa, small mammals act as reservoirs of *Borrelia* infections [3], [5]. *O. erraticus* and *O. sonrai* live inside burrows where they usually feed on rodents. However, they occasionally feed on humans by night, particularly when burrows open inside houses or when humans rest or sleep in the fields or the wild [5]. In Eastern and Southern Africa, there is no known mammal reservoir, and *O. moubata* and *O. porcinus*, that live in the cracks of the walls and grounds of human dwellings, act both as the vector and the reservoir of infection [4], [6], [7].

In Morocco, the presence of *Ornithodoros* ticks of the *O. erraticus* complex and the occurrence of TBRF in humans due to *B. hispanica* was established in the 1920s [8]–[11]. In 1937, a second spirochaete different from *B. hispanica* and *B. crocidurae* was isolated from *O. erraticus* s.l. in southern Morocco [12]. Initially considered as close to *B. duttoni* from East Africa, this spirochaete was later named *B. merionesi* [13]. Further studies in the 1950s indicated that populations of *O. erraticus* from different regions of Morocco and other parts of Africa were generally infertile in cross-mating experiments, and that those with the larger size (“grande forme” or “large variety”), classically described as *O. erraticus* s.s., transmitted *B. hispanica*, although the smaller ones (“petite forme” or “small variety”), morphologically attributable to *O. sonrai*, transmitted *B. crocidurae* or *B. merionesi* [14]–[16]. Both species were present in Morocco, with *O. erraticus* distributed in the wet areas of northern Morocco and *O. sonrai* in the arid southern part of the country, with sympatry of the two species in a dry intermediary area between Agadir and Marrakech [15]. There is little recent work on TBRF in Morocco. The disease has been reported from travellers [17] and a prospective study in six villages of northern Morocco indicated that 26 of 127 patients (20.5%) with an unexplained fever had TBRF attributable to *B. hispanica* [18].

Here we report a series of epidemiological investigations that we conducted between 2006 and 2011 on the distribution of vector ticks, the prevalence of *Borrelia* infection in ticks and small mammals, and the occurrence of the disease in patients.

## Materials and Methods

### *Ornithodoros* sampling

The study was carried out from September to October 2006, then from October to November 2009 and finally in May 2010 and November 2011 in 34 sites distributed in most regions of Morocco including the Atlantic Sahara. *Ornithodoros* ticks were sampled in diverse natural or human-impacted habitats. Ticks were collected by introducing a flexible tube into rodent or insectivore burrows - from 30 cm to 1.5 meter depth - and extracting their contents using a portable petrol-powered aspirator. As a general rule, 10 to 30 burrows were explored in each site in one or more sampling stations, selected according to local environment, either in natural or human-impacted ecosystems and croplands or both. All tick specimens collected were preserved in 95% ethanol. Identification of *Ornithodoros* ticks was performed according to classical morphological criteria [16], [19] and ticks of the *O. erraticus* complex were attributed either to *O. erraticus* or *O. sonrai* on the basis of the mean and maximum sizes of adult male and female specimens (lengths according to Baltazard [14]: 5.2 mm to 7.5 mm for *O. erraticus* females, 3.5 mm to 4.5 mm for *O. sonrai* females; 3.3 mm to 4.1 mm for *O. erraticus* males, 2.5 mm to 3.2 mm for *O. sonrai* males).

### Small mammal sampling

Rodents and insectivores were captured alive in November 2008 in ten sites using wire mesh traps baited with peanut butter or onions. Trapped rodents and insectivores were killed by means of cervical dislocation, and necropsied in the field. A thick blood film stained with Giemsa was prepared for microscopic examination and 200 oil-immersion fields (×1000) were systematically examined for detecting *Borrelia* spirochaetes. A sample of brain tissue was taken and preserved in 95% ethanol for PCR detection and identification of *Borrelia* species. Rodent and insectivore species were determined to species either morphologically or using karyological (standard karyotypes obtained from bone marrow or cell cultures) and molecular (cytochrome b gene sequence data analyses) methods, described in detail elsewhere [20].

### *Borrelia* detection

Each soft tick and brain tissue sample collected from small mammals was processed individually. They were washed in three sterile water baths (Versylene Fresenius, Sèvres, France), air dried and preserved in sterile microtubes. Samples were individually crushed by shaking with a bead beater (mixer mill MM301, Qiagen) and DNA was isolated and purified using the DNeasy Tissue extraction Kit (Qiagen). *Borrelia* detection on tick samples and brain tissue was based on nested PCR amplification of a 350 bp fragment of the flagellin gene (FLA). The amplification was performed using primers (Bfpad and Bfpdu for the first PCR, Bfpbu and Bfpcr for the second PCR) designed for *B. duttoni* [21]. Each PCR was performed in a 25 µl volume containing 5 µl 5× buffer (Promega), 2 mM MgCl_2_, 200 µM of each dNTP, 0.2 µM of each primer and 2.5 unit of GoTaq DNA polymerase (Promega). 3 µl of DNA template was added in the first reaction and 1 µl of the first amplified mix was added in the second reaction. Amplification cycles for the first PCR (FLA) consisted of an initial DNA denaturation step at 94°C for 3 min followed by 30 cycles of 40 sec at 94°C, 40 sec at 55°C, 40 sec at 72°C and a final extension step was carried out for 10 min at 72°C. For the second PCR (FLA), amplified cycles consisted of an initial DNA denaturation step at 94°C for 3 min followed by 30 cycles of 40 sec at 94°C, 40 sec at 51°C, 40 sec at 72°C and a final extension step was carried out for 10 min at 72°C. Agarose gel of 1.2% in TAE 0.5× buffer was prepared and solidified in a tank. The gel tray was then placed in a generator filled with TAE buffer. 10 µl of each PCR sample product mixed with 2 µl of envision (Amaresco) were placed in each well of the gel. A mixture of 5 µl of size marker and 2 µl of envision was introduced in the first well of the gel, and the generator was then switched to 100 volts, leaving bands to migrate by electrophoresis. The amplified products were detected after electrophoresis, and PCR results were observed in a trans-illuminator UV-tray. Amplified products were controlled in each nested PCR using a negative control of the second PCR and positive control for *Borrelia* to monitor contamination and false-positive amplification.

For the 16S–23S ribosomal RNA intergenic spacer (IGS), the primers rrs rrlA IGS/F and rrs rrlA IGS/R were performed for the first PCR, rrs rrlA IGS/Fn and rrs rrlA IGS/Rn for the second PCR [22]. Each PCR was performed in a 25 µl volume containing 5 µl 5× buffer (Promega), 2 mM MgCl_2_, 200 µM of each dNTP, 5 picomoles of each primer and 2.5 unit of GoTaq DNA polymerase (Promega). 2 µl of DNA template was added in the first reaction and 1 µl of the first amplified mix was added in the second reaction. Amplification cycles for the first PCR (IGS) consisted of an initial DNA denaturation step at 94°C for 3 min followed by 35 cycles of 30 sec at 94°C, 30 sec at 56°C, 30 sec at 72°C and a final extension step was carried out for 5 min at 72°C. For the second PCR (IGS), amplified cycles consisted of an initial DNA denaturation step at 94°C for 3 min followed by 40 cycles of 40 sec at 94°C, 30 sec at 60°C, 30 sec at 72°C and a final extension step was carried out for 5 min at 72°C. The amplified products were detected by electrophoresis as above. Amplified PCR products were controlled as described previously.

The FLA PCR products were sequenced and compared to *B. crocidurae* (Genbank acc. no. X75204), *B. duttoni* (Genbank acc. no. AB105128), *B. hispanica* (Genbank acc. no. U28498), *B. recurrentis* (Genbank acc. no. D86618). The IGS PCR products were sequenced and compared to *B. crocidurae* (Genbank acc. no. DQ000287) and *B. duttoni* (Genbank acc. no. DQ000279), *B. hispanica* (Genbank acc. no. FJ827590) and *B. recurrentis* (Genbank acc. no. DQ000277). A maximum-likelihood (PhyML) tree construction was based on the partial concatened FLA and IGS sequence data. All positions containing gaps and missing data were eliminated. There were a total of 694 positions in the final dataset and the evolutionary analysis was conducted in MEGA5 [23]. The *Borrelia* TARF1335 (*B. merionesi*), BERK1258 (*B. hispanica*) and BOUD1198 (*B. crocidurae*) were sampled from ticks of Tarfaya (Sidi Akhfennir), Berkane and Boudnib areas, respectively, and longitude and latitude of sampling sites are provided in Table 1. The Genbank accessions numbers for the IGS and FLA sequences were as follows: TARF1335 (IGS: Acc. no. JX257047), BERK1258 (IGS: Acc. no. JX257048), BOUD1198 (IGS: Acc. no. JX257049), TARF1335 (FLA: Acc. no. JX257050), BERK1258 (FLA: Acc. no. JX257051), and BOUD1198 (FLA: Acc. no. JX257052).

**Table 1:** Results of *Ornithodoros erraticus* s.l surveys in Morocco.

					N0. of collected	N0. infected ticks	*Borrelia*
Study area	Coordinate of	Date	Burrows		ticks	/N0. tested ticks	species +*
	sampling sites		Studied	With *O. erraticus* s.l (%)			
Tetouan	35°52′N/05°21′W	28/10/2009	10	7 (70%)	60	2/9	1
Izemmourèn	34°11′N/03°59′W	29/10/2009	15	4 (27%)	56	1/9	1
Berkane Oued Kiss	34°59′N/02°08′W	12/10/2006	30	15 (50%)	94	7/64	1
Ghouazi/Garba	35°01′N/06°08′W	12/05/2010	15	15 (100%)	298	1/5	1
Kenitra	34°18′N/06°29′W	10/10/2006	15	2 (13%)	6	0/5	0
Oued Ouerrha	34°34′N/06°04′W	30/10/2009	10	5(50%)	41	0/7	0
Ouled Ziane	34°33′N/06°22′W	31/10/2009	7	1 (14%)	2	0/2	0
Rabat	34°00′N/06°49′W	12/05/2010	15	13 (86.6%)	124	1/5	1
West Aiti Yadine	34°00′N/06°02′W	13/05/2010	15	10 (66.6%)	70	1/5	
Fes (Diamant Vert)	33°59′N/05°01′W	14/05/2010	15	9 (60%)	76	1/5	1
Beb-Lerba	34°00′N/04°05′W	15/05/2010	8	1 (12%)	12	0/5	0
Aïn-Benimathar	34°05′N/02°03′W	14/10/2006	30	13 (43%)	58	5/39	2
Oued Mellah	33°39′N/07°23′W	11/05/2010	15	12 (80%)	164	0/7	0
Bir-Jdid	33°22′N/08°00′W	23/10/2009	15	10 (67%)	78	1/10	1
Oued Oum Er-Rbiat	32°56′N/08°03′W	18/05/2010	15	12 (80%)	104	0/5	0
Oued Grou	32°55′N/06°02′W	13/05/2010	9	4 (44%)	22	0/5	0
Oued Choufcherk	33°02′N/04°00′W	16/05/2010	15	10 (67%)	141	0/5	0
Tendrara	33°01′N/02°00′W	14/10/2006	30	2 (7%)	4	0/2	0
Figuig	32°10′N/01°21′W	15/10/2006	10	3 (30%)	7	0/3	0
South-west Boudnib	31°59′N/03°59′W	17/10/2006	30	4 (13%)	2 4	4/23	2
Marrakech	31°44′N/07°58′W	26/10/2009	15	13 (87%)	175	1/15	2
Oued Tensift	32°00′N/09°20′W	24/10/2009	16	8 (50%)	59	0/14	0
Jbel Sarhro	30°56′N/05°50′W	18/10/2006	30	8 (27%)	25	0/8	0
Tata	29°53′N/08°05′W	07/10/2006	30	14 (47%)	35	3/29	2
Guelmim	29°03′N/09°55′W	06/10/2006	30	14 (47%)	51	5/33	3
Sidi Akhfennir	27°57′N/11°57′W	05/10/2006	30	15 (50%)	72	6/53	2; 3
Sidi Ahmed	26°51′N/11°56′W	04/10/2006	30	2 (7%)	2	0/2	0
North east Boujdour	26°13′N/14°20′W	01/10/2006	10	2 (20%)	2	0/2	0
South-east Bou Kra	25°58′N/12°52′W	03/10/2006	30	0	0	0	0
Galtat Zemmour	25°13′N/12°26′W	02/10/2006	20	2 (10%)	2	0/2	0
El Argoub	23°33′N/15°53′W	01/10/2006	10	5 (50%)	4	0/2	0
Aousserd	22°37′N/14°28′W	13/11/2011	12	1 (8%)	4	0/0	0
Lahmiris	22°04′N/16°35′W	14/11/2011	6	1 (17%)	3	0/0	0
Adrar Souttouf	21°51′N/15°29′W	16/11/2011	12	2 (17%)	5	0/0	0
**Total**			**605**	**239 (39.5%)**	**1, 880**	**39/382 (10.2%)**	

* : 1 = B. hispanica ; 2 = B. crocidurae ; 3 = B. merionesi.

### Clinical study

The study was conducted as part of the epidemiological monitoring of the risk of re-emergence of malaria in the Gharb plain (Kenitra, Souk El Arbaa, Souk Tlet El Gharg and Sidi Slimane medical districts, provinces of Kenitra and Sidi Slimane, northwestern Morocco), an area of 4,745 km^2^ with a population of 1,450,000 inhabitants (2004) which was, until the 1970s, a major focus of malaria. Outpatients with unexplained fever at 10 medical centers had thick blood films performed and sent to the Malaria Diagnosis Laboratory at the Provincial Hospital of Kenitra. From 2000 to 2005, besides the presence of *Plasmodium*, the presence of *Borrelia* was systematically investigated and reported in laboratory records including data on the age and gender of patients.

### Ethics Statement

Small mammal studies were conducted with authorization n°1369/08 of the Faculty of Sciences of the University Mohammed V (Rabat). Animals were treated in a humane manner, and in accordance with authorizations and guidelines of the American Society of Mammalogists (Animal Care and Use Committee 1998). Authorization for access to patient records was provided by the Malaria Diagnosis Laboratory at the Provincial Hospital of Kenitra and the Université Ibn Tofail of Kenitra. All records were anonymized. The study protocol was approved by the Steering Committee of the IRD Special Programme Evolution Climatique et Santé (Montpellier, France).

## Results

### *Ornithodoros* tick distribution

**Figure 1:**
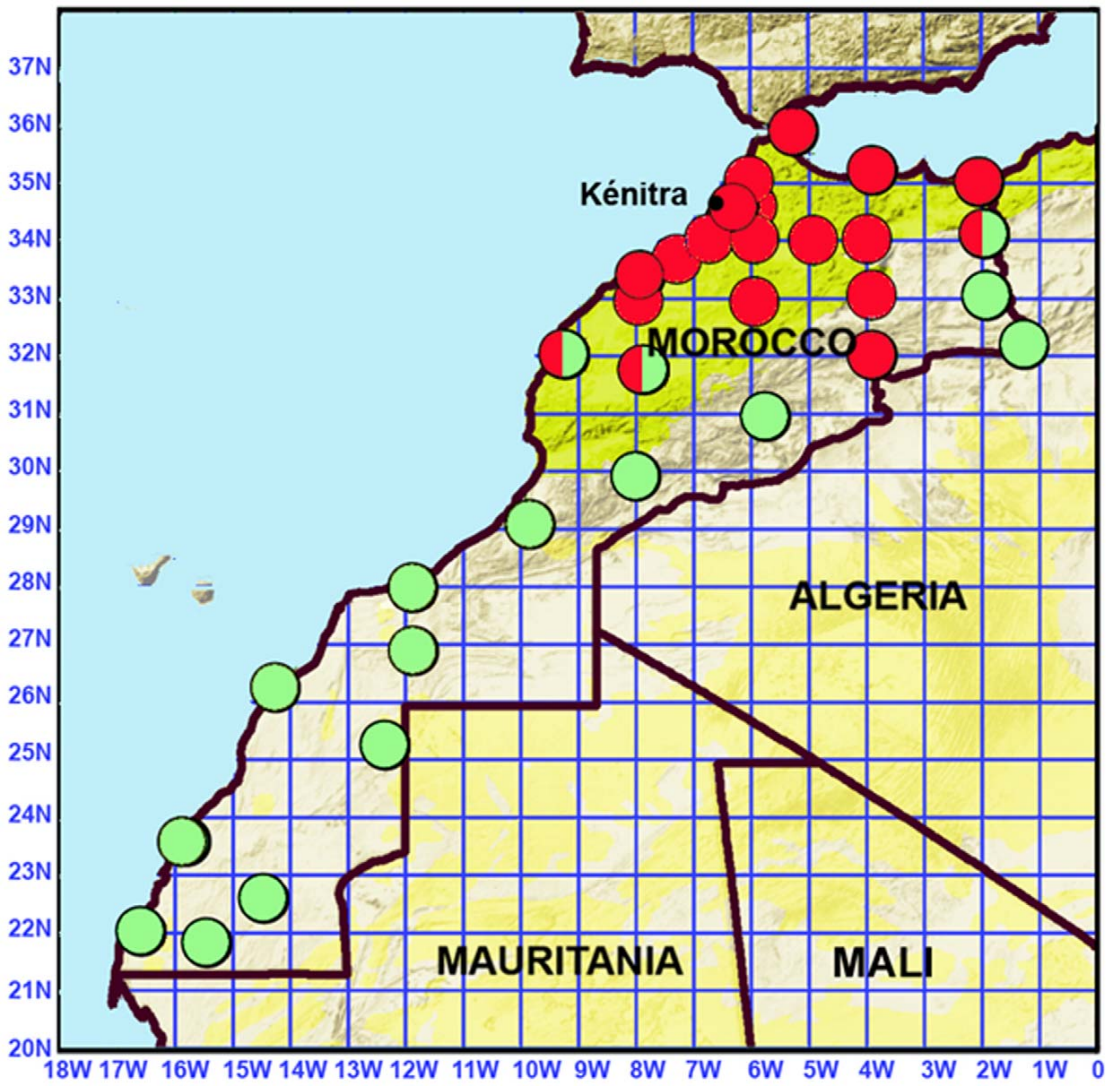
Geographic distribution of *Ornithodoros erraticus* s.s. (red circles) and *O. sonrai* (light green circles).

A total of 605 burrows of small rodents and insectivores were investigated in 34 sites. Argasid ticks of the *O. erraticus* complex were found in 39.5% (239/605) of burrows studied (Table 1). Among the 34 sites investigated, 33 were positive for *Ornithodoros* (Figure 1). The only negative site was a sandy desert area near Bou Kra where 30 burrows were examined at 25°58′N/12°52′W.

Within the *O. erraticus* complex, populations comprising only small specimens morphologically attributable to *O. sonrai* were distributed in southern and eastern Morocco, and those with larger specimens attributable to *O. erraticus* were distributed in the northwestern part of the country (Figure 1).

**Figure 2:**
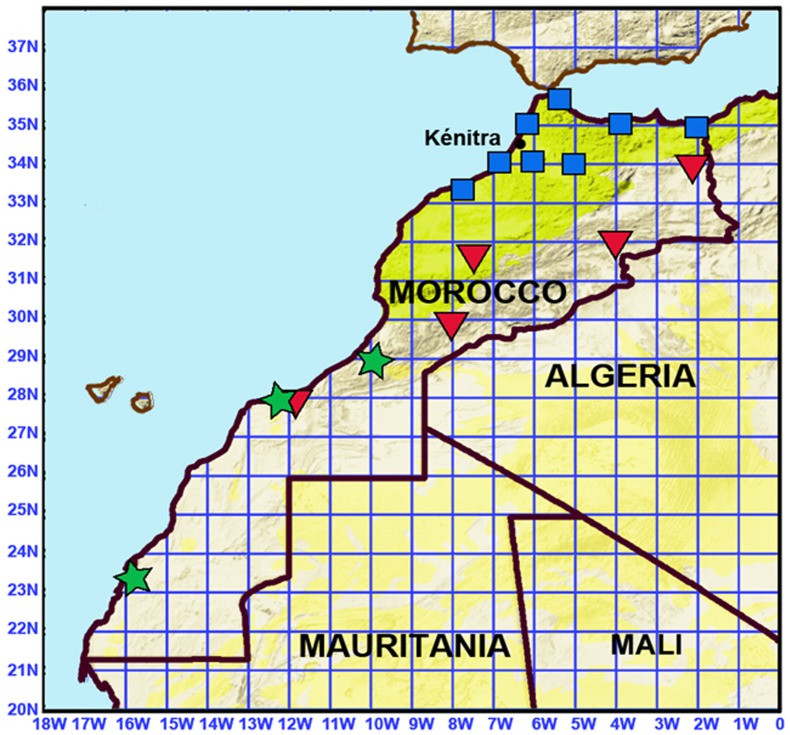
Geographic distribution of *Borrelia hispanica* (squares), *B. crocidurae* (triangles) and *B. merionesi* (stars) infections.

**Figure 3:**
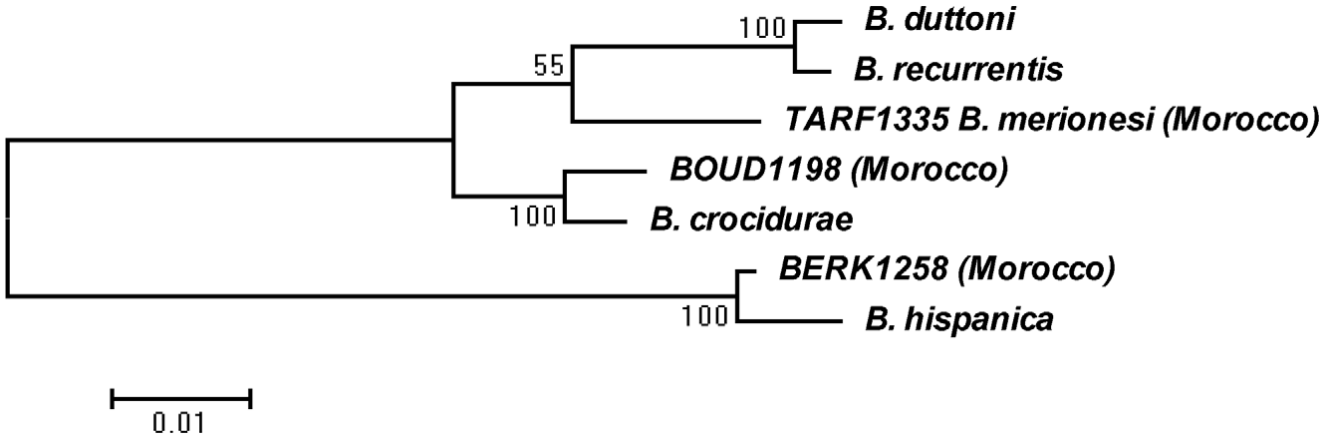
Phylogenetic relationships among *Borrelia* species in Morocco.

Of 382 *O. erraticus* s.l. ticks tested, 39 (10.2%) were infected by *Borrelia*: *B. hispanica* 13 (33.3%), *B. crocidurae* 16 (41%), and *B. merionesi* 10 (25.6%) (Figure 2). There was no significant difference in prevalence of infection among females (23% positive of 65 females tested) and males (32% positive of 50 males tested) in a sample of 110 adult ticks that were sexed, but prevalence was only 6% in a sample of 63 nymphs (p = 002 by Pearson's chi-squared test). Among the 239 burrows colonized by soft ticks, 159 had a sample of ticks tested, and 27 (17%) contained at least one *Borrelia* infected tick. The phylogenetic relationship (PhyML) between *Borrelia* species clearly shows the presence of three *Borrelia* species in *O. erraticus* s.l. ticks in Morocco (Figure 3).

### Small mammal study

A total of 140 small mammals including 134 rodents and 6 insectivores belonging to 12 different species were collected in 10 sites during 1,735 trap-nights of capture (Table 2). Out of 140 small mammals captured, 134 were tested for *Borrelia* by thick blood film and 140 via PCR of brain tissue (Table 3). The presence of *Borrelia* was observed in the blood of four rodents (3%) captured near Marrakech (two *Dipodillus campestris*), Essaouira (one *Meriones shawi*) and Taroudant (one *Gerbillus hoogstrali*). Out of the 140 animals whose brain tissue was tested by nested PCR amplification, *Borrelia* infections were detected in 12 specimens (8.6%) belonging to five species: 3 *Dipodillus campestris* and 2 *Meriones shawi* from Marrakech; 4 *Meriones sha*wi from Essaouira; 1 *Gerbillus hoogstrali*, 1 *Atelerix algirus*, and 1 *Gerbillus occiduus* respectively from Taroudant, Bou-Jerif and Dakhla.

**Table 2:** Sites of capture of small mammals.

Study area	Coordinates of sampling sites	Trapping effort (No of trap.nights)	Habitat sampled	Species captured (No. of specimens)
N of Marrakech	31°50′N/07°58′W	300	Farmlands	*Dipodillus campestris* (23), *Meriones shawi* (1)
S of Essaouira	31°30′N/09°46′W	400 (incl. 5 in the city)	Farmlands and city	*Dipodillus campestris* (20), *Meriones shawi* (8)
				*Rattus norvegicus* (2), *Mastomys erythroleucus* (1)
S of Taroudant	30°24′N/08°55′W	150	Orchards and farmlands	*Gerbillus hoogstrali* (12), *Meriones shawi* (2),
				*Dipodillus campestris* (4), *Meriones lybicus* (1) *Lemniscomys barbarus* (1)
Souss Massa	30°04′N/09°39′W	80	Gardens and dunes	*Dipodillus campestris* (1), *Gerbillus* sp. (2)
				*Mastomys erythroleucus* (1), *Atelerix algirus* (2)
N of Aglou	29°50′N/9°48′W	170	Natural coastal habitats	*Gerbillus* sp. (9), *Dipodillus campestris* (1)
				*Meriones shawi* (2), *Atlantoxerus getulus* (3)
				*Atelerix algirus* (3)
Fort Bou Jerif	29°04′N/10°20′W	145	Natural stony habitats	*Atelerix algirus* (1)
Aoreora	28°50′N/10°50′W	145	Natural sandy habitats	*Gerbillus occiduus* (8)
Between Tan-Tan and El Ouatia	28°29′N/11°14′W	50	Natural sandy habitats	*Gerbillus occiduus* (1)
N of Tarfaya	27°58′N/12°48′W	145	Natural coastal habitats	*Gerbillus occiduus* (6), *Gerbillus gerbillus* (10),
Dakhla	23°54′N/15°48′W	100	Dunes	*Gerbillus occiduus* (14), *Meriones shawi* (1)

**Table 3:** Prevalence of *Borrelia* infections in small mammals.

Species	N0. collected		N0. infected/N0. tested	
		Direct blood film	PCR from brain tissue	Any infection
*Dipodillus campestris*	49	2/49 (4%)	3/49 (6.1%)	4/49 (8.1%)
*Gerbillus hoogstrali*	12	1/12 (8.3%)	1/12 (8.3%)	1/12 (8.3%)
*Gerbillus gerbillus*	10	0/8	0/10	0/10
*Gerbillus* sp.	11	0/10	0/11	0/11
*Meriones libycus*	1	0/1	0/1	0/1
*Meriones shawi*	14	1/13 (7.6%)	6/14 (42.8%)	6/14 (42.8%)
*Atelerix algirus*	6	0/6	1/6 (16.6%)	1/6 (16.6%)
*Atlantoxerus getulus*	3	0/3	0/3	0/3
*Rattus norvegicus*	2	0/2	0/2	0/2
*Mastomys erythroleucus*	2	0/2	0/2	0/2
*Lemniscomys barbarus*	1	0/1	0/1	0/1
*Gerbillus occiduus*	29	0/27	1/29 (3.4%)	1/29 (3.4%)
**Total**	**140**	**4/134 (3%)**	**12/140 (8.6%)**	**13/140 (9.2%)**

Among the 134 animals studied by both methods, nine were positive by PCR alone, one by thick blood film only and three by both methods. *Borrelia* infections were not determined to species in small mammals in sites where *Borrelia* infections were previously determined to species in *Ornithodoros* ticks. In Dakhla area, where our small sample of ticks was negative, one *Gerbillus occiduus* out of 24 (4%) tested was found infected by *B. merionesi*.

### Borreliosis in clinic outpatients

**Figure 4:**
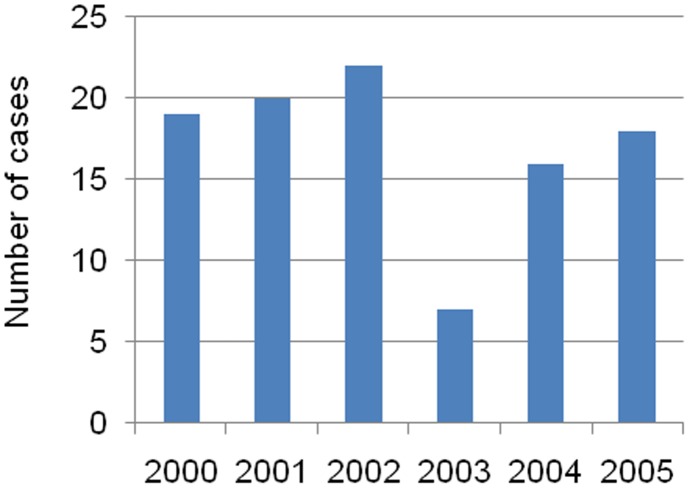
Annual distribution of TBRF cases in clinic outpatients.

**Figure 5:**
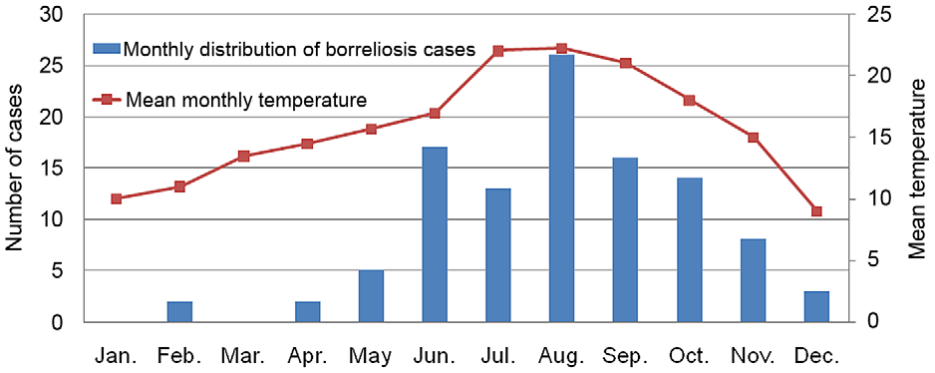
Monthly distribution of TBRF cases in clinic outpatients.

A total of 102 TBRF cases out of 89,995 patients examined (0.11%) were diagnosed between 2000 and 2005 (57% male and 43% female). No locally transmitted malaria case was observed. The annual number of TBRF cases ranged between 7 and 22 (Figure 4). All patients who were positive complained of acute fever. There was a marked seasonal pattern in the distribution of cases, most of them occurring between June and November (Figure 5). Patients aged 15 years or more represented 60% of TBRF cases and children less than one year, 1–4 years, 5–9 years and 10–14 years represented 3%, 7%, 12% and 18% of cases respectively.

## Discussion

Argasid ticks morphologically attributable to *O. erraticus* or *O. sonrai* were observed in high densities in almost all areas of Morocco, including the Atlantic coast of Sahara. Although the occurrence of these vectors in Morocco was well known [14]–[16], no detailed data on their relative abundance in burrows, and only limited distribution data within the country, were available. The widespread occurrence of TBRF vectors in the Sahara desert close to areas of Mauritania where we previously reported the presence of *O. sonrai* [24], [25] suggest a continuous distribution of this species between West and North Africa through the Sahara desert. In West Africa, this species has been reported from Mauritania, Senegal, Mali, and Gambia [1], [4]. Classical morphological criteria proved useful to identify soft ticks of the *O. erraticus* complex and to separate *O. sonrai* from *O. erraticus*. However, our recent studies in West Africa have shown molecular divergences within the *O. erraticus* complex [25], and this may also be the case in Morocco where the status of *O. marocanus*, a junior synonym of *O. erraticus*, appears uncertain [26]–[28]. The morphologically well defined *O. normandi* from northern Tunisia [29] was absent from our Moroccan samples.

Ten per cent of *Ornithodoros* ticks were found infected by *Borrelia* species and infected ticks were detected in half of the sites investigated. Since only a limited number of ticks was tested in most sites (often less than ten) and sites with infected ticks were documented in all regions of the country, this suggests that *Borrelia* infections among *Ornithodoros* ticks are widespread in Morocco. In a recent study in Senegal, the proportion of *O. sonrai* specimens infected by *B. crocidurae* averaged 31% and infected ticks were observed in all sites where the vector was collected [1]. Of the three species of *Borrelia* that we identified in Morocco, two are well known: *B. hispanica* is the species classically responsible for TBRF in Spain and North Africa, and previous molecular studies identified this species in patients with unexplained fever in northwestern Morocco [9], [18]; *B. crocidurae* is responsible for TBRF in West Africa [30], [31] and was also documented in *O. sonrai* ticks from southern Tunisia (*O. erraticus* small variety according to the authors of the study) [29]. The third species that we documented in *Gerbillus occiduus* from Dakhla and in *O. sonrai* ticks from Guelmin (formerly Goulimine) and Sidi Akhfennir, along the Saharan coast, was found in areas of southern Morocco where *Borrelia merionesi* (Blanc & Maurice, 1948) was first isolated in *Meriones shawi* in 1937. This *Borrelia* was recognized as a new species by these authors on the basis of difference in pathogenicity for laboratory animals and humans when compared to *B. hispanica*, *B. crocidurae* and *B. duttoni* [12], [13]. We attribute to this poorly known species the *Borrelia* infections observed in *Ornithodoros* ticks and in rodents living in this part of the Sahara desert bordering the Atlantic Ocean, an arid area characterized by presence of sea spray and dew. However, a few previous experiments with *B. merionesi* failed to induce TBRF in humans [12], [13].

Five different species of small mammals were identified as presumed reservoirs for *Borrelia* infections in one or more of the nine areas that we investigated, suggesting that they may play an important role in the epidemiology of TBRF in Morocco. To our knowledge, there is no previous published data on the prevalence of *Borrelia* infections in small mammals in North Africa. As previously observed in Senegal, both rodents and insectivores were found to be infected [5]. The proportion of infected animals in our study was 9.2%, a value higher than the 2.3% prevalence rate reported from Mauritania [24], but lower than those observed in Senegal where prevalence varied between 12% to 33% according to studies [5], [32], [33]. The detection of *Borrelia* infections in small mammals depends of the method used and the organ tested, and testing brain may be more sensitive than testing blood [33].

Clinical data show that TBRF is a common disease in Morocco. The Gharb region where the clinical study took place was an important focus of malaria until the 1970s. Thick blood films are made in outpatient clinics in case of unexplained fever as part of a surveillance program for preventing the reemergence of malaria. *Borrelia* infections are frequently diagnosed by trained microscopists but they are not systematically reported. Reports were unfortunately interrupted since 2006 for some obscure reason. The 102 TBRF cases documented in a single area over a 6 year period confirm that the disease is common and that all age groups are affected. Since our study was retrospective, *Borrelia* infections in these patients were not genotyped, however all infections previously investigated in this area were found to be due to *B. hispanica* [18].

Monthly distribution of cases shows a strong seasonality of the disease during the warmest period of the year. This may reflect both increased activity of the vector ticks during this period and increased exposure of the population.

*Ornithodoros* ticks of the *O. erraticus* complex live inside burrows where they feed on rodents and insectivores, but they occasionally go outside the burrows by night in order to feed on other animals or humans [5]. In contrast to West Africa where almost all rural populations live in houses, where rodent burrows are numerous and often open in bedrooms, this is rarely the case in Morocco, both in urban and rural areas. The proportion of TBRF cases among patients with fever is often comprised between 2% and 10% of cases in Senegal [1], [31] although it was only 0.11% in Morocco. Risk factors were not investigated in our study, but farming activities, camping and recreational activities likely represent important risk factors. Epidemiological surveys among TBRF patients are needed to have a better understanding of the modalities of contamination. Further work is also needed, to map the tick/borrelia distributions in relation to environmental and climatic characteristics.

### List of accession numbers (Genbank)

*B. merionesi TARF1335* : JX257047 (IGS)

*B. merionesi TARF1335*: JX257050 (FLA)

*B. hispanica BERK1258*: JX257048 (IGS)

*B. hispanica BERK1258*: JX257051 (FLA)

*B. hispanica*: FJ827590 (IGS)

*B. hispanica*: U28498 (FLA)

*B. crocidurae BOUD1198*: JX257049 (IGS)

*B. crocidurae BOUD1198*: JX257052 (FLA)

*B. crocidurae*: DQ000287 (IGS)

*B. crocidurae*: X75204 (FLA)

*B. recurrentis*: DQ000277 (IGS)

*B. recurrentis*: D86618 (FLA)

*B. duttoni*: DQ000279 (IGS),

*B. duttoni*: AB105128 (FLA)
